# Multi-center, randomized, double-blind, placebo-controlled study of quetiapine extended-release formulation in Japanese patients with bipolar depression

**DOI:** 10.1007/s00213-018-4977-6

**Published:** 2018-08-01

**Authors:** Mitsukuni Murasaki, Tsukasa Koyama, Shigenobu Kanba, Masahiro Takeuchi, Yuriko Shimizu, Eri Arita, Kentaro Kuroishi, Masahiro Takeuchi, Shinya Kamei

**Affiliations:** 1 Institute of CNS Pharmacology, 3-14-20 Sagamiohno, Minami-ku, Sagamihara, Kanagawa 252-0303 Japan; 2 Ohyachi Hospital, Clinical Research Center, 5-7-10 Ohyachi-higashi, Atsubetsu-ku, Sapporo, Hokkaido 004-0041 Japan; 3 Department of Neuropsychiatry, Graduate School of Medical Sciences, Kyushu University, 3-1-1 Maidashi, Higashi-ku, Fukuoka, 812-8582 Japan; 4 Department of Clinical Medicine, School of Pharmacy, Kitasato University, 5-9-1 Shirokane, Minato-ku, Tokyo, 108-8641 Japan; 5 Japan/Asia Clinical Development 2, Astellas Pharma Inc., Astellas Pharma Inc.; 2-5-1 Nihonbashi-Honcho, Chuo-ku, Tokyo, 103-8411 Japan; 6 Global Clinical Science, Astellas Pharma Europe B.V, Sylviusweg 62, PO Box 344, 2300 AH Leiden, The Netherlands; 7 Japan-Asia Data Science, Astellas Pharma Inc., 2-5-1 Nihonbashi-Honcho, Chuo-ku, Tokyo, 103-8411 Japan; 8 Astellas Pharma Global Development, Inc., 1 Astellas Way, Northbrook, IL 60062 USA

**Keywords:** Atypical antipsychotics, Quetiapine XR, Bipolar disorder, Depression, MADRS

## Abstract

**Rationale:**

Quetiapine fumarate is an atypical antipsychotic indicated for various mental disorders, but it has not been studied in Japanese patients with bipolar depression.

**Objectives:**

To evaluate the efficacy and safety of quetiapine XR (extended release) in Japanese patients with bipolar depression.

**Methods:**

In this multi-center, randomized, double-blind, placebo-controlled, fixed-dose study of 431 Japanese adults with bipolar I or II disorder, efficacy was determined by analyzing the mean change from baseline in Montgomery-Åsberg Depression Rating Scale (MADRS) total score. Secondary end points included MADRS response and remission rates, Hamilton Depression Scale 17-Item (HAM-D_17_), and Clinical Global Impressions-Bipolar (CGI-BP) scale scores. Safety was determined by monitoring adverse events and clinical assessments.

**Results:**

This study revealed a statistically significantly greater decrease in MADRS total score after 8 weeks of quetiapine XR 300 mg/day monotherapy compared with placebo (− 12.6 vs. − 10.1; *p* = 0.034). There were also improvements in MADRS response (44.1 vs. 35.6%) and remission (38.0 vs. 26.6%) rates as well as in HAM-D_17_ and CGI-BP scale scores compared with placebo. In the subgroup analysis of patients with bipolar I or II disorder, the adjusted mean changes in MADRS total score compared to placebo were − 2.3 and − 2.1, respectively. Adverse events occurred in 149 patients (83.2%) receiving quetiapine XR 300 mg/day and in 81 patients (45.8%) receiving placebo. The most common adverse events were somnolence and thirst, which is consistent with the previously reported safety profile.

**Conclusions:**

Once-daily monotherapy with quetiapine XR is an effective and well-tolerated treatment for bipolar depression in Japanese patients.

## Introduction

Bipolar disorder is a mental illness involving at least one manic or hypomanic episode. Most patients experience at least one major depressive episode, though some only experience manic episodes (American Psychiatric Association 2013). Bipolar disorder is a spectrum of unstable emotional conditions that includes bipolar I disorder and bipolar II disorder. The estimated lifetime prevalence of bipolar I and II disorders in Japan is 0.1% for both disorders (Merikangas et al. 2011).

Quetiapine, an atypical antipsychotic drug with dopamine D_2_ receptor and serotonin 5-HT_2A_ receptor antagonizing activity (Gefvert et al. 1998; Saller and Salama 1993), has been approved and used worldwide for various mental disorders, such as schizophrenia and bipolar disorder. The antidepressant activity of quetiapine may be mediated, at least in part, by its metabolite norquetiapine through noradrenaline transporter inhibition and partial 5-HT_1A_ receptor agonism (Jensen et al. 2008). Confirmatory studies of quetiapine immediate-release (IR) and extended release (XR) tablets have shown that quetiapine is effective and safe for bipolar disorder patients in a depressive state (Calabrese et al. 2005; McElroy et al. 2010; Suppes et al. 2010; Thase et al. 2006; Young et al. 2010). In these studies, the primary outcome was to measure the change in the Montgomery-Åsberg Depression Rating Scale (MADRS) total score from baseline to week 8. For both quetiapine IR and XR 300 mg/day, there was a statistically significantly greater change in MADRS total score than in the placebo group. The most common reported adverse events (AEs) in the quetiapine group were somnolence and dry mouth.

Despite the fact that quetiapine monotherapy is recommended as one of the first-line treatments for bipolar depression in several treatment guidelines (Goodwin et al. 2016; Grunze et al. 2010; Kanba et al. 2013; Yatham et al. 2013), its efficacy and safety have not been studied in Japanese patients. In Japan, quetiapine IR has been approved only for schizophrenia but is routinely used off label for patients with bipolar depression. Therefore, the development of quetiapine XR has been initiated for major depressive episodes in bipolar patients, who are considered to have high medical needs. Because quetiapine XR is administered once a day, better adherence rates are expected when compared with quetiapine IR, which requires multiple administrations per day.

This was a multi-center, randomized, double-blind, controlled study aimed at evaluating the efficacy and safety of quetiapine XR 150 or 300 mg after 8 weeks of oral administration in Japanese patients with bipolar depression. Patients who completed the 8-week double-blind study were invited to take part in an open-label extension study, the results of which will be reported separately.

## Methods

### Study design

Written informed consent was obtained from all patients prior to participation. Ethical approval was provided by the Institutional Review Board at each participating site.

This was a multi-center, randomized, double-blind, placebo-controlled, parallel group comparative trial across 98 sites in Japan, initiated in February 2012 and completed August 2015. The study consisted of an 8-week double-blind trial in patients diagnosed with bipolar I disorder or bipolar II disorder.

**Fig. 1:**
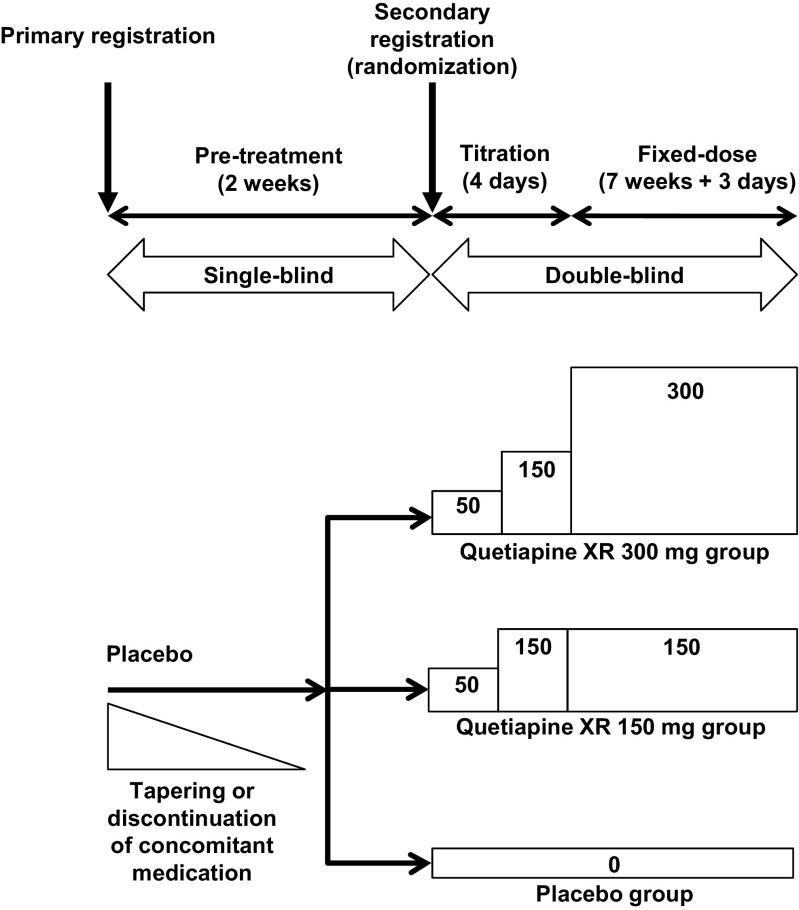
Study design

Patients received placebo for the first 2 weeks in a single-blind manner. Subsequently, patients received quetiapine XR treatment or placebo for 8 weeks, including a dose-titration period. The study design is shown in Fig. 1. The randomization was stratified by bipolar diagnosis (bipolar I disorder or bipolar II disorder).

### Patient population

Eligible patients were aged 20 to 64 years; had a documented clinical diagnosis meeting the Diagnostic and Statistical Manual of Mental Disorders, 4th Edition, Text Revision (DSM-IV-TR) criteria for bipolar I disorder or bipolar II disorder; a recent depressive episode (296.50 to 296.54 or 296.89) as confirmed by Mini-International Neuropsychiatric Interview (M.I.N.I) (American Psychiatric Association 2000); a Hamilton Depression Scale 17-Item (HAM-D_17_) total score ≥ 20 points and a HAM-D_17_ depressed mood score ≥ 2 points; and a negative pregnancy test result in female patients of childbearing potential.

Patients who met any of the following criteria were not eligible for participation: concurrent or previous history of DSM-IV-TR Axis I disorders, except for bipolar disorder, within 6 months prior to informed consent; concurrent DSM-IV-TR Axis II disorder considered to greatly affect the patient’s current mental status; a Young Mania Rating Scale (YMRS) total score of 13 points or more; nine or more mood episodes within 12 months prior to informed consent; a lack of response with at least two different antidepressants for more than 6 weeks; a current major depressive episode exceeding 12 months or less than 4 weeks prior to informed consent; a history of substance or alcohol abuse; a HAM-D_17_ suicide score of 3 points or more; or a history of suicide attempt within 6 months prior to informed consent.

### Study medication

Patients were initially randomized in a 1:1:1 ratio to receive 150 mg/day quetiapine XR, 300 mg/day quetiapine XR, or placebo. Patients received quetiapine XR or matching placebo orally once daily at bedtime for 8 weeks. Patients in the quetiapine XR 150 mg group were started on an initial dose of 50 mg/day and titrated to 150 mg/day on the third day of treatment (day 3). In the quetiapine XR 300 mg group, patients were started on an initial dose of 50 mg/day and titrated to 150 mg/day on day 3 and 300 mg/day on day 5. After re-evaluating the study feasibility due to the difficulty in recruiting a sufficient number of patients in a timely manner, and after consultation with the Pharmaceuticals and Medical Devices Agency, the decision was made to discontinue the assignment of patients to the quetiapine XR 150 mg group. Patients randomized to the quetiapine XR 150 mg group continued receiving 150 mg/day quetiapine XR after the discontinuation of further patient assignment to this group.

### Prior and concomitant medications

The following drugs were not allowed for concomitant use, except for those specified as conditionally allowed concomitant drugs: mood stabilizers (lithium carbonate, sodium valproate), lamotrigine, antipsychotics, antidepressants, antiepileptics, antianxiety agents, hypnotics, sedatives, cytochrome P450 3A4 (CYP3A4) inhibitors or inducers, monoamine oxidase inhibitors, psychostimulants, antiparkinsonian agents, cerebral ameliorators, antidementia agents, anorectics, or adrenaline.

Conditionally allowed concomitant drugs were as follows: lorazepam (if it had been used for at least 14 days before the primary registration), only one hypnotic (zopiclone, triazolam, or eszopiclone, which had been used for at least 14 days before the primary registration), and only one anticholinergic (indicated for the treatment of extrapyramidal symptoms).

### Efficacy evaluations

The primary efficacy end point was the change from baseline to week 8 (end of treatment) in the MADRS total score.

Secondary end points were MADRS response (defined as patients whose MADRS total score decreased by 50% or more from baseline), MADRS remission (defined as patients whose MADRS total score was ≤ 12), HAM-D_17_, Clinical Global Impressions-Bipolar (CGI-BP)-Severity of Illness (-S), and Change (-C) scores. A CGI-BP-C rating of “much improved” or “very much improved” defined patients as having a CGI-BP-C response.

Clinical assessments of MADRS and HAM-D_17_ were conducted at the primary registration (visit 1), at the secondary registration on day 1 (visit 2, baseline), at weeks 1, 2, 3, 4, 6, and 8 (visits 3–8), and at follow-up (week 10, if not proceeding to the extension study). CGI-BP-S was determined at visits 2–8 and follow-up, and CGI-BP-C was determined at visits 3–8 and follow-up.

### Safety and tolerability

Safety variables included AEs, laboratory assessments (hematology, blood biochemistry, and urinalysis), body weight, vital signs (blood pressure and pulse rate), 12-lead electrocardiography (ECG) with QT interval and corrected QT (QTc), Drug-Induced Extra Pyramidal Symptoms Scale (DIEPSS), YMRS, and the Columbia Suicide Severity Rating Scale (C-SSRS).

### Statistical analyses

The target sample size at the beginning of the study considered multiplicity and was based on previous quetiapine clinical trials (Calabrese et al. 2005; McElroy et al. 2010; Suppes et al. 2010; Thase et al. 2006; Young et al. 2010). Assuming a difference of 4.0 units in MADRS total score from baseline to the end of treatment at week 8 between groups with a combined standard deviation (SD) of 11.3, the required number per group was determined to be 200 patients. Discontinuation of patient assignment to the quetiapine XR 150 mg group was accompanied by a change to the multiplicity adjustment. The target sample size was recalculated and adjusted to 170 patients in each of the remaining two groups.

The full analysis set (FAS) was defined as the efficacy analysis population and included patients who had been treated with the study drug at least once and for whom measurements were taken for at least one variable pertaining to efficacy after the start of treatment. The set of patients who were treated with the study drug at least once were also included in the safety analysis set.

The primary efficacy analysis was the change in MADRS total scores from baseline compared with placebo using an analysis of covariance model including baseline as the covariate and treatment group and bipolar disorder diagnosis as fixed effects. To complement the missing data, the last observation carried forward (LOCF) approach was used. To evaluate the robustness of the primary analysis, mixed effect models for repeated measures (MMRM) was used as the secondary analysis. Regarding the efficacy results of the 150 mg quetiapine XR group, assignment to the group was stopped during the study; therefore, these results were handled as reference data only.

## Results

### Patient disposition

**Fig. 2:**
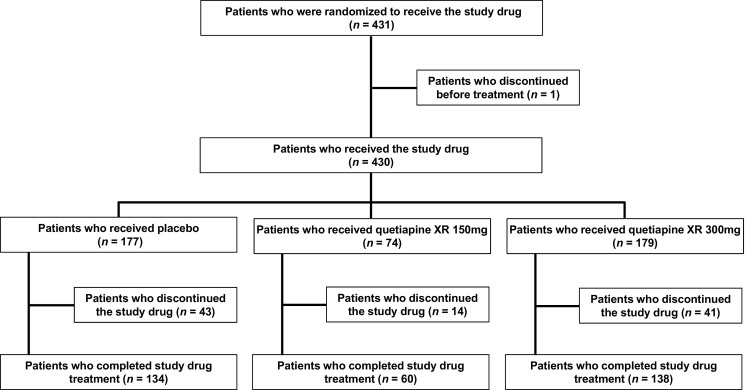
Patient disposition

Of the 659 patients who gave written informed consent, 431 met the inclusion criteria and were randomized to the study. One of these patients discontinued before treatment; therefore, the study drug was administered to 179 patients in the 300 mg quetiapine XR group, 74 in the 150 mg quetiapine XR group, and 177 patients in the placebo group (Fig. 2). A total of 332 patients completed treatment: 138 in the quetiapine XR 300 mg group, 60 in the quetiapine XR 150 mg group, and 134 in the placebo group. The demographic and clinical characteristics of the treatment groups were well matched and are shown in Table 1.

**Table 1:** Demographic and clinical characteristics of patients enrolled in the study

Variable		Placebo group (*n* = 177)		Quetiapine XR 150 mg group (*n* = 74)		Quetiapine XR 300 mg group (*n* = 179)	
Age (years), mean (SD)		38.8	(11.0)	39.2	(10.2)	38.1	(11.2)
Sex, *n* (%)	Male	76	(42.9%)	37	(50.0%)	86	(48.0%)
	Female	101	(57.1%)	37	(50.0%)	93	(52.0%)
Diagnosis, *n* (%)	Bipolar I disorder	51	(28.8%)	22	(29.7%)	51	(28.5%)
	Bipolar II disorder	126	(71.2%)	52	(70.3%)	128	(71.5%)
No. of mood episodes, *n* (%)	≥ 4	13	(7.3%)	4	(5.4%)	14	(7.8%)
MADRS total score, mean (SD)		30.8	(6.4)	30.2	(6.8)	30.9	(6.9)
HAM-D_17_ total score, mean (SD)		23.1	(2.8)	23.3	(3.4)	23.0	(3.0)

*MADRS* Montgomery-Åsberg Depression Rating Scale, *HAM-D*_*17*_ Hamilton Depression Scale 17-Item, *SD* standard deviation, *XR* extended release

### Efficacy

#### MADRS

The mean (SD) change from baseline in the MADRS total score at the end of treatment (week 8) was − 10.1 (10.9) in the placebo group and − 12.6 (11.4) in the quetiapine XR 300 mg group (Table 2). The difference in the adjusted mean of the change from baseline between the quetiapine XR 300 mg group and the placebo group was − 2.4 (95% confidence interval [CI] − 4.7, − 0.2), and the decrease in the quetiapine XR 300 mg group was statistically significantly greater than that in the placebo group (*p* = 0.034). In the MMRM analysis, the difference in the adjusted mean between the quetiapine XR 300 mg group and the placebo group at week 8 was − 2.4 (95% CI − 4.7, − 0.0), and the difference was statistically significant (*p* = 0.049).

**Table 2:** Change from baseline in MADRS total score and HAM-D_17_ total score

			Difference from placebo^a^	
	Placebo group (*n* = 177)	Quetiapine XR 300 mg group (*n* = 179)	LS mean (two-sided 95% CI)	*P* value
MADRS total score				
Baseline	30.8 (6.4)	30.9 (6.9)	–	–
End of treatment	20.6 (11.9)	18.2 (11.2)	–	–
Change from Baseline	− 10.1 (10.9)	− 12.6 (11.4)	− 2.4 (− 4.7, − 0.2)	0.034
HAM-D_17_ total score				
Baseline	23.1 (2.8)	23.0 (3.0)	–	–
End of treatment	14.7 (8.3)	12.9 (7.1)	–	–
Change from Baseline	− 8.4 (7.6)	− 10.1 (7.6)	− 1.7 (− 3.3, − 0.1)	0.033

Mean (SD)

*MADRS* Montgomery-Åsberg Depression Rating Scale, *HAM-D*_*17*_ Hamilton Depression Scale 17-Item

^a^Analysis of covariance (ANCOVA) including treatment and bipolar diagnosis (bipolar I disorder or bipolar II disorder) as fixed factors and baseline as a covariate

**Fig. 3:**
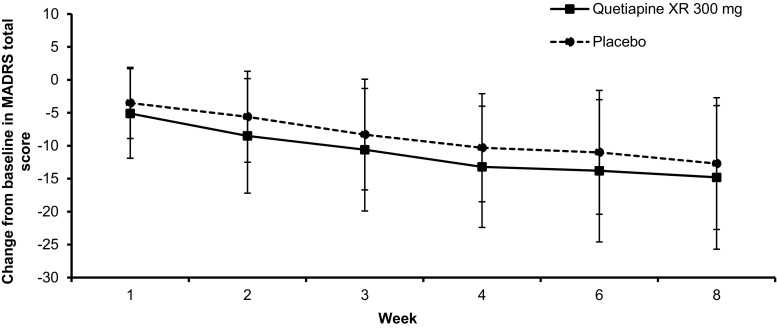
Change from baseline in MADRS total score after treatment with quetiapine XR 300 mg or placebo for 8 weeks. Error bars depict standard deviations

The time course of change from baseline in the MADRS total score (mean, SD) is presented in Fig. 3. The mean (SD) MADRS total score was 30.8 (6.4) in the placebo group, 30.9 (6.9) in the quetiapine XR 300 mg group at baseline, and 20.6 (11.9) in the placebo group and 18.2 (11.2) in the quetiapine XR 300 mg group at the end of treatment. The mean MADRS total score in the quetiapine XR 300 mg group was lower than that in the placebo group at any time point. The mean (SD) MADRS total score in the quetiapine XR 150 mg group was 30.2 (6.8) at baseline and 15.8 (8.8) at the end of treatment point, and the mean (SD) change from baseline was − 14.4 (11.4) (data not shown).

The proportions of patients with a MADRS response or remission are shown in Table 3. The proportion of patients with a MADRS response increased over time in both groups and at the end of treatment was higher in the quetiapine XR 300 mg group (44.1%) than in the placebo group (35.6%). The proportion of patients with a MADRS remission also increased over time in both groups and at the end of treatment was higher in the quetiapine XR 300 mg group (38.0%) than in the placebo group (26.6%).

**Table 3:** Proportions of patients with MADRS response, MADRS remission, and CGI-BP-C response at the end of treatment

		Placebo group (*n* = 177)	Quetiapine XR 300 mg group (*n* = 179)
Patients with a response based on MADRS total score		63 (35.6%)	79 (44.1%)
Patients with a remission based on MADRS total score		47 (26.6%)	68 (38.0%)
Patients with a response based on CGI-BP-C score	Overall bipolar illness	64 (36.2%)	77 (43.0%)
	Depression	65 (36.7%)	77 (43.0%)
	Mania	1 (0.6%)	2 (1.1%)

*n* (%)

*MADRS* Montgomery-Åsberg Depression Rating Scale, *CGI-BP-C* Clinical Global Impressions-Bipolar-Change

A subgroup analysis on the basis of bipolar diagnosis (bipolar I disorder or bipolar II disorder) was performed when assessing the change from baseline in the MADRS total score. The differences in the adjusted mean change from baseline between the quetiapine XR 300 mg group and the placebo group in patients with bipolar I disorder and bipolar II disorder were − 2.3 and − 2.1, respectively. Subgroup analyses for sex, age, MADRS total score at baseline, and HAM-D_17_ total score at baseline were also performed for the change from baseline in the MADRS total score. Overall, a greater decrease in the MADRS total score was seen in the quetiapine XR 300 mg group than in the placebo group regardless of subgroup. However, in the group with HAM-D_17_ total scores of < 22 at baseline, the decrease in the MADRS total score was greater in the placebo group than in the quetiapine XR 300 mg group.

#### HAM-D_17_

The change from baseline in the HAM-D_17_ total score at the end of treatment is shown in Table 2. The mean (SD) change from baseline in the HAM-D_17_ total score was − 8.4 (7.6) in the placebo group and − 10.1 (7.6) in the quetiapine XR 300 mg group. The difference in the adjusted mean of the change from baseline between the quetiapine XR 300 mg group and the placebo group was − 1.7 (95% CI − 3.3, − 0.1), and the decrease in the quetiapine XR 300 mg group was statistically significant compared with the placebo group (*p* = 0.033).

#### CGI-BP-S

The mean (SD) CGI-BP-S (overall bipolar illness, depression) score was 4.5 (0.7), 4.5 (0.7), respectively, in the placebo group; 4.4 (0.8), 4.5 (0.7), respectively, in the quetiapine XR 300 mg group at baseline; 3.5 (1.4), 3.5 (1.4), respectively, in the placebo group; and 3.2 (1.2), 3.2 (1.2), respectively, in the quetiapine XR 300 mg group at the end of treatment. The mean (SD) change from baseline at the end of treatment was − 1.0 (1.3), − 1.0 (1.4), respectively, in the placebo group and − 1.2 (1.3), − 1.2 (1.3), respectively, in the quetiapine XR 300 mg group.

#### CGI-BP-C

The proportion of patients with a response based on CGI-BP-C (overall bipolar illness, depression) increased over time both in the placebo and the quetiapine XR 300 mg groups but was higher in the quetiapine XR 300 mg group (43.0, 43.0%, respectively) than in the placebo group (36.2, 36.7%, respectively) at the end of treatment (Table 3).

### Safety and tolerability

#### AEs

A summary of the AEs that occurred in either group is provided in Table 4. The incidence of AEs was higher in the quetiapine XR 300 mg group (83.2%) than in the placebo group (45.8%), and the incidence of drug-related AEs was also higher in the quetiapine XR 300 mg group (74.3%) than in the placebo group (29.4%).

**Table 4:** Summary of adverse events (AEs) and AEs occurring in at least 5% of the patients in any of the treatment groups

	Placebo group (*n* = 177)		Quetiapine XR 300 mg group (*n* = 179)		Quetiapine XR 150 mg group (*n* = 74)	
	Patients *n* (%)	Number of AEs	Patients *n* (%)	Number of AEs	Patients *n* (%)	Number of AEs
AEs	81 (45.8)	155	149 (83.2)	432	55 (74.3)	144
Drug-related AEs	52 (29.4)	88	133 (74.3)	331	50 (67.6)	107
Deaths	1 (0.6)	0	0	0	0	0
SAEs	2 (1.1)	2	0	0	1 (1.4)	1
Drug-related SAEs	0	0	0	0	0	0
AEs leading to discontinuation	16 (9.0)	17	27 (15.1)	35	6 (8.1)	8
Drug-related AEs leading to discontinuation	11 (6.2)	12	23 (12.8)	30	5 (6.8)	7
	Patients (*n*)	Patients (%)	Patients (*n*)	Patients (%)	Patients (*n*)	Patients (%)
GI disorders	22	12.4	34	19.0	15	20.3
Constipation	3	1.7	14	7.8	10	13.5
General disorders and administration site conditions	10	5.6	61	34.1	12	16.2
Malaise	2	1.1	16	8.9	3	4.1
Thirst	5	2.8	50	27.9	9	12.2
Infections and infestations	22	12.4	38	21.2	15	20.3
Nasopharyngitis	19	10.7	26	14.5	12	16.2
Investigations	16	9.0	41	22.9	15	20.3
ALT increased	0	0	8	4.5	4	5.4
AST increased	1	0.6	2	1.1	4	5.4
Blood creatine phosphokinase increased	1	0.6	5	2.8	7	9.5
Blood prolactin increased	5	2.8	13	7.3	4	5.4
Nervous systems disorders	17	9.6	95	53.1	29	39.2
Akathisia	4	2.3	15	8.4	7	9.5
Dizziness	1	0.6	5	2.8	4	5.4
Somnolence	4	2.3	80	44.7	26	35.1
Psychiatric disorders	20	11.3	12	6.7	2	2.7
Depression	10	5.6	4	2.2	1	1.4

*AEs* adverse events, *SAEs* serious adverse events, *GI* gastrointestinal, *ALT* alanine aminotransferase, *AST* aspartate aminotransferase

The incidence of AEs during the treatment period that resulted in discontinuation was 15.1% (27/179 patients) and 9.0% (16/177 patients) in the quetiapine XR 300 mg group and placebo group, respectively. In this study, quetiapine XR was titrated to a daily dose of 300 mg in 5 days and was well tolerated in most patients. The most common AE observed in the quetiapine XR 300 mg group was somnolence (44.7% in the quetiapine XR 300 mg group and 2.3% in the placebo group), followed by thirst (27.9% in the quetiapine XR 300 mg group and 2.8% in the placebo group) (Table 4).

The severity of AEs during the treatment period was predominantly mild or moderate. Two severe AEs (completed suicide, depression) in the placebo group and one severe AE (retinal detachment) in the quetiapine XR 150 mg group were reported. Of these, completed suicide and retinal detachment were considered as SAEs. Other SAEs included anaphylactic reaction in one patient in the placebo group.

The incidence of AEs related to manic or hypomanic symptoms was higher in the placebo group (2.3%) compared with the quetiapine XR 300 mg group (1.1%). The most common AE related to manic or hypomanic symptoms was hypomania with an incidence of 2.3% in the placebo group and 0.6% in the quetiapine XR 300 mg group. Patients had low YMRS total scores at baseline, and the mean (SD) YMRS total score was 1.9 (1.5) in the placebo group and 2.1 (1.7) in the quetiapine XR 300 mg group. There was no worsening of mean (SD) YMRS total scores in either the placebo group or the quetiapine XR 300 mg group [1.7 (2.3) vs. 1.2 (2.1), respectively] at the end of treatment.

The incidence of AEs related to extrapyramidal symptoms was higher in the quetiapine XR 300 mg group (12.3%) compared with the placebo group (4.0%). The most common extrapyramidal symptom was akathisia, with an incidence of 2.3% in the placebo group and 8.4% in the quetiapine XR 300 mg group. There was no significant difference between the placebo group and the quetiapine XR 300 mg group in drug-induced extrapyramidal symptoms as assessed by DIEPSS at the end of the treatment.

The incidence of AEs related to suicide was 2.3% in the placebo group (suicide attempt [1.1%], completed suicide [0.6%], and intentional self-injury [0.6%]) and 2.2% in the quetiapine XR 300 mg group (self-injurious behavior [1.1%], intentional self-injury [0.6%], and suicidal ideation [0.6%]).

#### Clinical laboratory evaluations

Changes in clinical laboratory evaluations are presented in Table 5. No marked changes in systolic or diastolic blood pressure were found in either the placebo group or the quetiapine XR 300 mg group during the study. The mean change (SD) from baseline in body weight was − 0.54 (1.96) and 0.92 (2.35) kg in the placebo group and the quetiapine XR 300 mg group, respectively.

**Table 5:** Changes in clinical laboratory evaluations

	Baseline						End of treatment						Change from baseline					
	Placebo group		Quetiapine XR 300 mg group		Quetiapine XR 150 mg group		Placebo group		Quetiapine XR 300 mg group		Quetiapine XR 150 mg group		Placebo group		Quetiapine XR 300 mg group		Quetiapine XR 150 mg group	
	Mean	SD	Mean	SD	Mean	SD	Mean	SD	Mean	SD	Mean	SD	Mean	SD	Mean	SD	Mean	SD
Body weight (kg)	62.65	13.10	63.15	13.71	64.09	13.06	62.32	13.37	64.16	13.92	64.33	12.87	− 0.54	1.96	0.92	2.35	0.45	2.27
Blood glucose (mg/dL)	97.9	15.8	98.5	14.2	96.8	13.9	98.4	16.3	101.3	17.1	97.1	12.3	0.5	19.2	3.1	18.1	0.3	15.8
HbA1c (%)	5.01	0.28	5.05	0.27	5.07	0.28	5.03	0.29	5.06	0.31	5.06	0.32	0.01	0.15	0.01	0.17	− 0.01	0.24
Total cholesterol (mg/dL)	195.1	39.9	191.0	35.6	192.7	40.8	191.2	33.4	195.5	37.5	193.8	36.1	− 4.1	23.3	4.9	26.0	1.1	28.2
HDL-C (mg/dL)	58.1	14.4	56.5	16.7	55.3	13.1	57.8	14.1	55.7	16.3	54.4	13.5	− 0.2	8.2	− 0.7	8.0	− 0.9	9.0
LDL-C (mg/dL)	117.2	37.4	115.4	32.0	116.9	36.3	115.3	31.3	118.4	32.3	119.1	31.2	− 2.3	21.5	3.2	18.7	2.2	22.8
Triglycerides (mg/dL)	136.4	109.8	128.2	80.1	126.8	92.8	121.6	86.2	147.0	112.4	139.3	106.6	− 15.1	87.8	18.9	91.3	12.5	62.5
Prolactin (ng/mL)	11.983	15.409	10.529	6.779	9.044	4.491	12.433	20.655	12.375	19.305	10.300	5.286	0.375	23.310	1.877	19.479	1.256	4.504

*HbA1c* hemoglobin A1c, *HDL-C* high-density lipoprotein-cholesterol, *LDL-C* low-density lipoprotein-cholesterol

There were no clinically significant changes in the mean values of any of the hematological parameters. Regarding blood biochemistry parameters, the mean change from baseline in blood glucose levels and hemoglobin A1c were 3.1 mg/dL and 0.01%, respectively, in the quetiapine XR 300 mg group compared with 0.5 mg/dL and 0.01%, respectively, in the placebo group. The mean change from baseline in blood prolactin was 1.877 ng/mL in the quetiapine XR 300 mg group compared with 0.375 ng/mL in the placebo group. The mean change in triglyceride levels from baseline was 18.9 mg/dL in the quetiapine XR 300 mg group compared with − 15.1 mg/dL in the placebo group. The mean change in total cholesterol levels from baseline was 4.9 mg/dL in the quetiapine XR 300 mg group compared with − 4.1 mg/dL in the placebo group. However, for both triglyceride and total cholesterol levels, the observed increases at the end of treatment were still within the reference ranges.

Investigation of the 12-lead ECG findings revealed that two patients in the quetiapine XR 300 mg group had clinically significant abnormalities as judged by an investigator. One patient had a clinically insignificant abnormality (atrioventricular block first degree) at baseline, but a clinically significant abnormality (atrial flutter) at week 8 that was assessed as a mild AE. One other patient had clinically significant abnormalities (right axis deviation and atrioventricular block first degree) in the pre-treatment observation period that were also found at week 8 of the treatment period; however, this was not considered as an AE. None of the patients in either group had QTc (Fridericia) exceeding 480 ms at any assessment point with a mean (SD) change from baseline in QTc (Fridericia) of 1.8 (12.9) ms in the placebo group and 0.6 (11.8) ms in the quetiapine XR 300 mg group at the end of treatment.

## Discussion

This study was a double-blind, placebo-controlled, parallel group comparative study in patients diagnosed with major depressive episodes in bipolar disorder. The efficacy of quetiapine IR and quetiapine XR for the treatment of bipolar depression has been previously shown in several studies across different countries; however, this is the first study to evaluate the efficacy and safety of a quetiapine XR in Japanese patients with bipolar depression.

We demonstrated that a fixed dose of 300 mg quetiapine XR once daily is an effective monotherapy for the treatment of Japanese patients with bipolar depression. This was primarily evidenced by the statistically significantly greater decrease in the MADRS total score from baseline in patients treated with quetiapine XR 300 mg after 8 weeks compared with the placebo group. The proportion of patients with a MADRS response and a MADRS remission increased over time, beginning as early as week 1, which suggests a rapid onset of action. Furthermore, in the MMRM analysis, the difference between the quetiapine XR 300 mg group and the placebo group at week 8 was statistically significant, which supports the primary analysis findings.

In the Guidelines for the Clinical Evaluation of Antidepressants, the change in HAM-D_17_ total score is also a recommended efficacy assessment measure (Pharmaceuticals and Medical Devices Agency in Japan 2010). In this study, the difference in the adjusted mean of the change from baseline in HAM-D_17_ was statistically greater for the quetiapine XR 300 mg treatment group compared with the placebo group. Quetiapine XR 300 mg treatment was also suggested to be more effective than placebo according to CGI-BP-S and CGI-BP-C scores. While CGI-BP-S and CGI-BP-C collectively allow a general assessment of patients’ clinical symptoms, they were also included because of their use as secondary end points in previously published placebo-controlled studies of quetiapine. These CGI-BP-S and CGI-BP-C ratings are consistent with previous clinical trial observations (Calabrese et al. 2005; McElroy et al. 2010; Suppes et al. 2010; Thase et al. 2006; Young et al. 2010). Furthermore, after 8 weeks, the proportion of patients with improvements in secondary end points, including HAM-D_17_ and CGI-BP scores, indicates that quetiapine XR monotherapy has a robust and consistent antidepressive effect.

When assessing patients’ depressive mood, the mean changes in MADRS item 1 (apparent sadness) and HAM-D_17_ item 1 (depressed mood) scores showed greater decreases in the quetiapine XR 300 mg group (− 1.4 and − 1.3, respectively) than in the placebo group (− 1.3 and − 1.0, respectively).

This study enrolled patients that were diagnosed with either bipolar I disorder or bipolar II disorder with depressive episodes. Previous results have shown that quetiapine IR and XR monotherapy have clinical efficacy in both bipolar I and II disorders in non-Japanese populations (Calabrese et al. 2005; McElroy et al. 2010; Suppes et al. 2010; Thase et al. 2006; Young et al. 2010). Subgroup analysis, when stratified by diagnosis, shows that quetiapine XR monotherapy is clinically effective in Japanese patients indicated for either bipolar I or II disorder, although the proportion of patients with bipolar II disorder that were included in this study was generally higher than that in most other quetiapine IR and XR studies.

In an exploratory analysis of the data, the numbers needed to treat (95% CI) was calculated to be 12 (< − 47, > 6) patients to achieve a MADRS response and 9 (5, 81) patients to achieve a MADRS remission (Add-hoc analysis).

AEs were observed in both treatment groups and were predominantly mild or moderate in severity. AEs such as somnolence, thirst, malaise, and dizziness were reported in the quetiapine XR 300 mg group, which is consistent with previous clinical trials for both quetiapine IR and quetiapine XR formulations (Gao et al. 2008; Muneer 2015; Suttajit et al. 2014). Other AEs related to extrapyramidal symptoms, such as akathisia, were observed in the quetiapine XR 300 mg group. The most common AE in the placebo group was nasopharyngitis (10.7%), followed by depression (5.6%), the latter of which was higher than in the quetiapine XR 300 mg group (2.2%).

In this study, the incidence of AEs related to manic or hypomanic symptoms was relatively low in the quetiapine XR 300 mg group compared with the placebo group. The treatment of bipolar depression with antidepressants comes with a known risk of switching to hypomania or mania (Leverich et al. 2006). However, in the present study, little manic switching occurred in the quetiapine XR 300 mg group compared with the placebo group, which is consistent with previous findings in a double-blind placebo-controlled trial of quetiapine XR (Suppes et al. 2010).

The abnormal clinical laboratory findings (weight increased, blood prolactin increased, and blood triglyceride levels) that were observed in this study are consistent with the safety profile of quetiapine. Patients treated with quetiapine XR reported an increase in body weight; however, this was not directly responsible for patient withdrawal from the study. Atypical antipsychotic-induced weight increase is often associated with abnormal changes to lipid metabolism, which is linked to an increase in cardiovascular disease risk. In the quetiapine XR group, higher mean changes in blood lipid variables were noted; however, the changes were not clinically meaningful and were within the range of previous reports. We also observed a higher, albeit clinically insignificant, mean change in blood glucose levels from baseline to end of treatment after quetiapine XR treatment.

The levels of blood prolactin tended to increase from baseline in the quetiapine XR 300 mg group compared with the placebo group, but this did not lead to study discontinuation for any patient. An increase in blood prolactin levels has previously been reported for a range of typical and atypical antipsychotics, in comparison to quetiapine (Gorobets 2005).

Although the proportions of AEs leading to discontinuation and drug-related AEs leading to discontinuation were greater in the treatment group compared with the placebo group (15.1 vs. 9.0% and 12.8 vs. 6.2%, respectively), patient adherence to the study shows that the study drug was well tolerated.

The present study has some limitations. It was a placebo-controlled, double-blind, parallel-group comparative trial; however, after re-evaluation, the allocation to the quetiapine XR 150 mg group was discontinued, and the study design was accordingly modified to evaluate the efficacy and safety of quetiapine XR at a dose of 300 mg, compared with placebo. Because patient randomization to the quetiapine XR 150 mg/day group was discontinued, this group could not be directly compared with other treatment groups. Additionally, the study was conducted in Japanese patients with bipolar depression, aged 20–64 years, limiting the generalizability of the findings to other populations.

In conclusion, the efficacy and safety of 300 mg/day quetiapine XR monotherapy, in comparison to placebo, in Japanese patients with bipolar depression were confirmed. A significant reduction in depressive symptoms was observed. AEs to therapy were reported; however, these have previously been reported in Japan and abroad, with no new safety concerns identified.
